# Variation vs. specialization: the dose-time-effect of technical and physiological variety in the development of elite swimmers

**DOI:** 10.1186/s13104-024-06706-x

**Published:** 2024-02-14

**Authors:** Dennis-Peter Born, Jenny Lorentzen, Glenn Björklund, Thomas Stöggl, Michael Romann

**Affiliations:** 1 Swiss Swimming Federation, Section for High-Performance Sports, Bern, Switzerland; 2 Department for Elite Sport, Swiss Federal Institute of Sport Magglingen, Hauptstrasse 247, 2532 Magglingen, Switzerland; 3 Computing in Science, University of Hamburg, Hamburg, Germany; 4 Swedish Winter Sports Research Centre, Mid Sweden University, Östersund, Sweden; 5 Red Bull Athlete Performance Center, Thalgau, Austria

**Keywords:** Competitive swimming, Deliberate practice, Diversification, Long-term athlete development, Sampling, Talent

## Abstract

**Objective:**

It is heavily discussed whether larger variety or specialization benefit elite performance at peak age. Therefore, this study aimed to determine technical (number of different swimming strokes) and physiological (number of different race distances) variety required to become an international-class swimmer (> 750 swimming points) based on 1′522′803 race results.

**Results:**

Correlation analyses showed lower technical variety in higher ranked swimmers (*P* < 0.001), yet with small effects (0.11–0.30). However, Poisson distribution revealed dose-time-effects and specified number of swimming strokes required during each age group. Specifically, freestyle swimmers showed highest chances when starting to compete in three to four swimming strokes but reduced their variety to three swimming strokes at the ages of 12/13yrs with another transition to two swimming strokes at the ages of 19/21yrs (female/male swimmers, respectively). Although both sexes showed similar specialization pattern throughout their career, earlier specialization was generally evident in female compared to male swimmers. At peak performance age, freestyle was most frequently combined with butterfly. Swimmers who either kept competing in all five swimming strokes or focused on only one at the beginning of their careers showed lowest probability of becoming an international-class swimmer. Physiological variety increased during junior age but declined again to three race distances towards elite age.

**Supplementary Information:**

The online version contains supplementary material available at 10.1186/s13104-024-06706-x.

## Introduction

During adolescence, participation in a large variety of sporting activities could improve success chances at peak performance age [1]. Additionally, late specialization and a broad development of motor skills delay performance plateaus and reduce risk of burnout and overuse injuries [2]. A recent review showed that successful elite age athletes specialized later and accumulated “less coach-led main-sport practice, but more other-sports practice” [3]. However, most participants in these studies that highlighted the value of late specialization and large variety played game sports, which allow for large skill transfer between different sports [4]. On the other hand, the relationship between accumulated hours of structured practice and increased level of expertise [5, 6] may be of particular importance to swimmers. As such, humans are evolutionary unaccustomed to water and technical adaptations may require more practice compared to land-based sports: firstly, water provides no solid resistance for swimmers to push off from, which reduces propelling efficiency, while at the same time the density of water increases drag forces [7]. Therefore, swimmers acquire twice the years of practice (estimated at about 1000 h per year [5, 8]) to reach world-class level compared to land-based team sports [9, 10], in which skill transfer from other sports are also more likely [9].

Although, swimmers may have to spend more time in their specific competition environment, i.e. water, swimmers may still benefit from a large variety of sport-specific practice, by including all swimming strokes, i.e. freestyle (FR), breaststroke (BR), backstroke (BA), and butterfly (BU), and their combination in a single event, i.e. individual medley (IM), in their competition schedule. Specific technical elements, i.e. undulating kicking (BU, FR, BA), catch at the beginning of the arm stroke (FR and BU), body rotation and core stability along the longitudinal body axis (FR and BA) may be more pronounced in some swimming strokes but still transferable to others. Additionally, race distances from highly anaerobic (50 m sprint) to long aerobic (1500 m) events may help to develop a large physiological variety, which may especially benefit middle-distance events that rely on both anaerobic and aerobic energy contribution [11].

Pool swimming competitions that are officially licensed by World Aquatics (World Swimming Federation) are little affected by meteorological (wind and precipitation) and standardized for thermoregulatory factors (water temperature of 25–28 °C), pool length (from + 0.010 m to − 0.000 m), and current (< 1.25 m/min) [12]. Consequently, swimming race results provide robust data for scientifically standardized analyses. Therefore, the aims of the study were to (1) investigate the relationship between performance level and degree of specialization, and (2) quantify technical (number of different swimming strokes) and physiological variety (number of different race distances) required to become an international-class swimmer (> 750 swimming points).

## Material and methods

### Subjects

For the present study, 1′522′803 race results of n = 4094 swimmers (2190 female and 1904 male) were included. The data were acquired from the database of the European Swimming Federation LEN (Ligue Européenne de Natation) [13] with permission for publication of the scientific results. The study was approved by the institutional review board of the Swiss Federal Institute of Sport Magglingen (registration number: 177_LSP_102022) and conducted in accordance with the Declaration of Helsinki. No consent for participation was required, as race results were anonymous and analyzed ex post facto from a publicly available database.

### Data analysis

First, all swimmers who still competed at peak performance age in any of the five 200 m long-course events were extracted from the database, and their personal best times at peak performance age were used to rank the swimmers for each event. Peak performance ages for FR (22/24), BR (24/24), IM (22/25), BA(22/25), and BU (22/24) were specified according to previous research for female and male swimmers, respectively [14]. The 200 m race results were used, as this is the only Olympic race distance that includes all swimming strokes and their combination in a single event, i.e. IM. As swimmers typically compete in long-course races (50 m pool length) at the season’s main competition, only long-course races were considered to establish the ranking at peak performance age. Next, all these swimmers’ race times from 10 to 26yrs of age were retrospectively extracted. As all swimming competitions contribute to the development process, both short- (25 m pool length) and long-course individual events were considered here. Finally, number of different swimming strokes (FR, BR, BA, BU) or their combination in a single event (IM) and number of different race distances (50 m, 100 m, 200 m, 400 m, 800 m, 1500 m) were filtered for each year of the swimmers’ careers. The data were handled with the Pandas library (Data Analysis and Manipulation Tool for Python, version 1.5.1, pandas-dev/pandas, Zenodo, Genève, Switzerland) for Python (version 3.9.7, Python Software Foundation, Beaverton, USA).

### Statistical analysis

In the initial stage of the data assessment, trends were explored using *correlation analyses*. Specifically, the relationship between performance level at peak performance age and the number of different swimming strokes and race distances competed in during development from early junior to elite age were examined. Age categories were used according to previous research [15] to represent the initial stage of competitive swimming (early junior age [10–14 years]), age at international junior competitions, i.e. European and World Junior Championships (late junior age [15–17 years]), transition from junior and elite competitions (transition age [18–20yrs]), and age of peak performance (elite age [21–26yrs] [14]). The Pearson’s correlation coefficient was replaced by Spearman’s if Q-Q plot showed non-normally distributed data. Statistical significance was accepted at an alpha level of 0.05.

In the next stage of the data assessment, the dose-time-effect of technical and physiological variety across the development process and the probability (*p*) of becoming an international-class swimmer was calculated using *Poisson distribution*. In accordance to previous guidelines, swimmers were considered international-class if they reached > 750 swimming points at peak performance age [16]. Swimming points were calculated according to the official method of the World Swimming Federation [12]. Poisson distribution was calculated based on number of swimming strokes (1–5) and race distances (1–6) using Microsoft Excel (365 MSO, version 2209, Microsoft Corporation, Redmond, WA, USA). Poisson distribution is a discrete probability mass function that shows the likelihood of an independent event, i.e. becoming an international-class swimmer at peak performance age, for multiple time points on a constant time scale. The likelihood is determined in percentages depending on the number of swimming strokes and race distances competed in during a particular year [17]. All other statistical analyses were conducted with JASP statistical software package version 0.16.4 (JASP-Team, University of Amsterdam, Amsterdam, The Netherlands).

## Results

### Technical variety

*Correlation analysis* revealed that higher ranked female swimmers of all stroke specializations competed in fewer swimming strokes after 15yrs of age (Table 1). In male swimmers, this lower technical variety was most pronounced in 200 m FR (*P* < 0.001), BR (*P* < 0.001), and BU (*P* < 0.05) but not IM (*P* > 0.05).

**Table 1:** Performances at peak age in freestyle, breaststroke, individual medley, backstroke, and butterfly were correlated with number of different swimming strokes and race distances used at competitions during the various age categories

Performance at peak age	Age groups [years]			
	10–14	15–17	18–20	21–26
Females				
Number of different swimming strokes (technical variety)				
Freestyle	− 0.15***	− 0.22***	− 0.22***	− 0.24***
Breaststroke	− 0.04	− 0.14*	− 0.21***	− 0.20***
Individual Medley	− 0.05	− 0.12***	− 0.12***	− 0.16***
Backstroke	− 0.11*	− 0.19***	− 0.23***	− 0.25***
Butterfly	− 0.19***	− 0.27***	− 0.30***	− 0.24***
Number of different race distances (physiological variety)				
Freestyle	0.07	0.06	0.09**	0.07*
Breaststroke	0.12	− 0.03	− 0.03	− 0.05
Individual Medley	0.07	− 0.05	− 0.05	− 0.07*
Backstroke	0.09*	− 0.08	− 0.17***	− 0.16***
Butterfly	− 0.06	− 0.23***	− 0.22***	− 0.20***
Males				
Number of different swimming strokes (technical variety)				
Freestyle	0.02	− 0.12***	− 0.16***	− 0.16***
Breaststroke	0.05	− 0.25***	− 0.19***	− 0.28***
Individual Medley	0.05	− 0.07	0.04	0.05
Backstroke	0.03	− 0.14*	− 0.08	− 0.02
Butterfly	0.04	− 0.16**	− 0.14*	− 0.22***
Number of different race distances (physiological variety)				
Freestyle	0.14***	0.10**	0.17***	0.15***
Breaststroke	0.11	− 0.09	− 0.03	− 0.05
Individual Medley	0.17**	0.09*	0.16***	0.17***
Backstroke	0.08	− 0.03	0.00	0.08
Butterfly	0.07	− 0.08	− 0.05	− 0.15**

Statistical significance: * *P* < 0.05; ** *P* < 0.01; *** *P* < 0.001

**Fig. 1:**
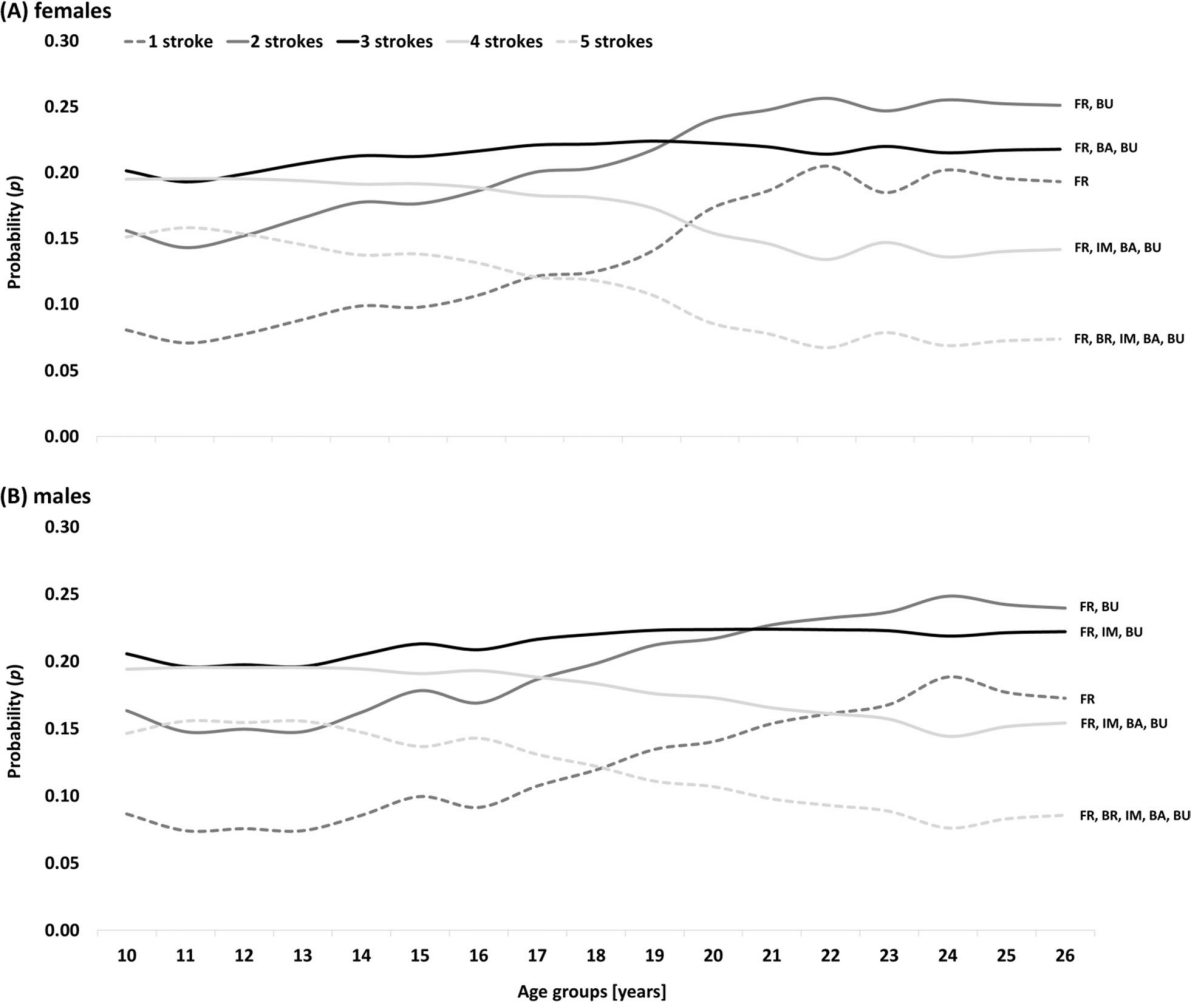
Probability (*p*) to become an international-class **A** female and **B** male *freestyle* swimmer (> 750 swimming points at peak performance age) when competing over different *numbers of swimming strokes* across the age groups. The text boxes on the right end of the graphs show the most frequent combinations of swimming strokes at peak performance age

*Poisson distribution* showed highest probability of becoming a female international-class FR swimmer when starting to compete in either three or four swimming strokes, ideally transitioning to three and two swimming strokes at the ages of 12 and 19yrs, respectively (Fig. 1). Male FR swimmers showed a similar pattern, except for the transition to three and two swimming strokes occurring later at 13 and 21yrs of age, respectively. In both female and male swimmers, competing in only one or all five swimming strokes showed low probabilities to become an international-class swimmer at early junior and elite age, respectively.

Compared to FR swimmers, BR, BA, and BU swimmers showed lower technical variety beginning with three swimming strokes during junior age (refer to Additional file 1: Figures S1–S4). Higher technical variety was evident in IM of both sexes beginning with four during early junior age and focusing on three swimming strokes at 17 and 19yrs of age for female and male swimmers, respectively. At peak performance age, BR was most frequently combined with IM. BA and BU were most frequently combined with FR.

### Physiological variety

*Correlation analysis* revealed that higher ranked female BU and BA swimmers at peak performance age were physiologically more specialized from the ages of 15 and 18yrs, respectively (*P* < 0.001). In contrast, higher ranked male FR and IM swimmers showed more physiological variety from the beginning of their career (*P* < 0.05, refer to Table 1).

**Fig. 2:**
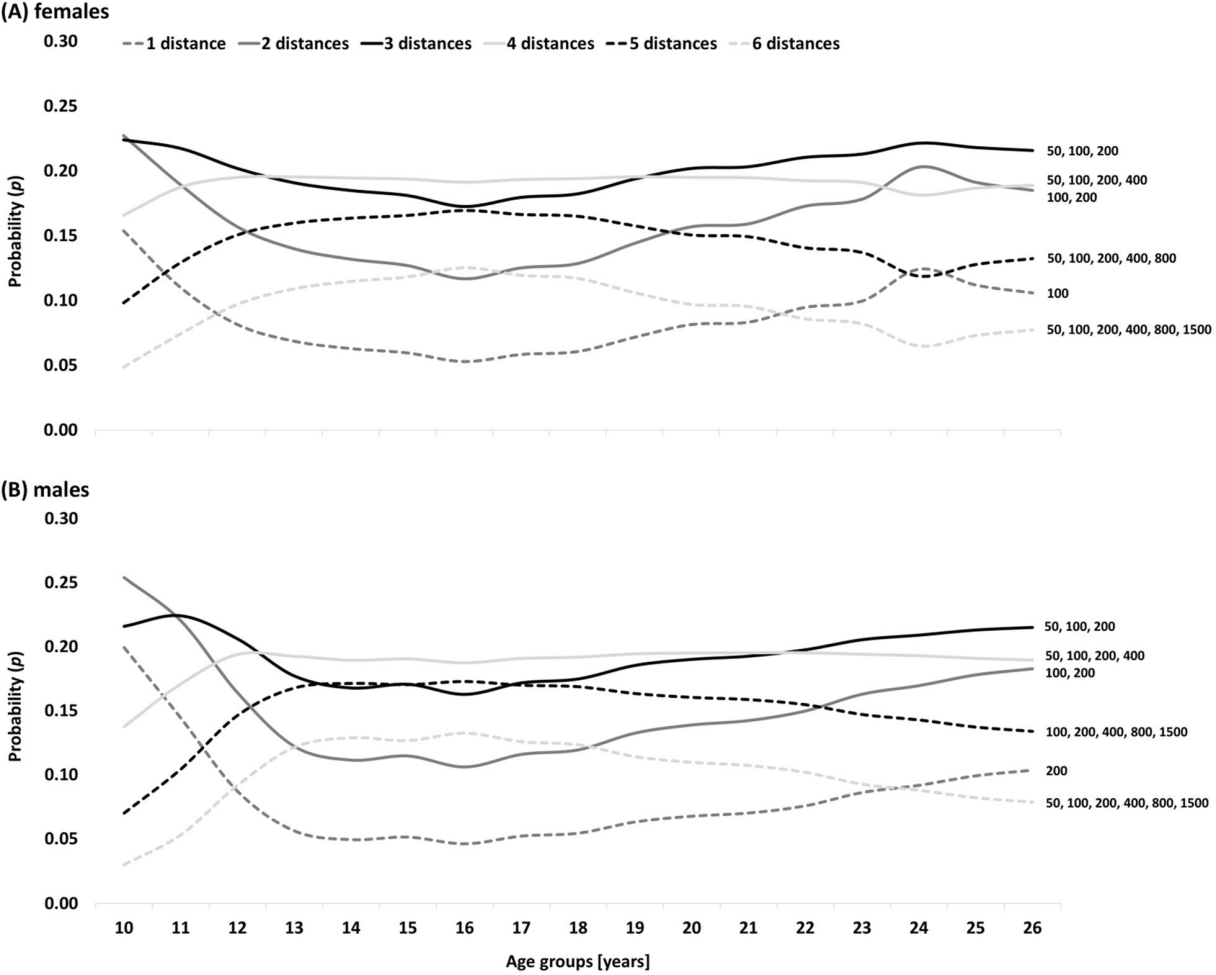
Probability (p) to become an international-class **A** female and **B** male *freestyle* swimmer (> 750 swimming points at peak performance age) when competing over different *numbers of race distances* across the age groups. The text boxes on the right end of the graphs show the most frequent combinations of race distances at peak performance age

*Poisson distribution* showed that starting at early junior age with two to three race distances leads to the highest probability of becoming a female international-class FR swimmer (*p* > 20%, Fig. 2). During late junior age (15-17yrs), three to five race distances were most advantageous. However, over five race distances, probability of becoming an international-class swimmer began to decline at 16yrs of age, with an ideal transition from four to three race distances at 19yrs of age. Male international-class FR swimmers showed a similar pattern but respond later. Probability of becoming an international-class swimmer with five race distances declined from 17yrs of age. Ideal transition from four to three race distances occurred at 22yrs of age. Competing over only one race distance showed low probability of becoming an international-class swimmer. Competing over six race distances reduced probability towards elite age. The dose-time-effect of BR, IM, BA, and BU is provided in the (Additional file 1: Figures S1–S4).

## Discussion

Correlation analyses showed that there was a significantly greater specialization in higher ranked swimmers, yet with a small effect. More specifically, Poisson distribution revealed the dose-time-effects for female FR swimmers, who should ideally start competing in three to four swimming strokes and then reduce variety to three and two swimming strokes at the ages of 12 and 19yrs (male swimmers: 13 and 21yrs), respectively. Generally, female swimmers started to specialize earlier than their male counterparts. At peak performance age, FR was most frequently combined with BU. Swimmers who kept competing in all five swimming strokes or focused on only one from the beginning of their career showed the lowest probabilities of becoming an international-class swimmer. Comparing the swimming strokes, BR, BA, and BU showed greater technical specialization, while IM showed greater technical variety than FR. Highest probability of becoming an international-class swimmer was evident for female FR swimmers with a physiological variety of three to five race distances during young age groups, while the benefit of five race distances began to decline from 16yrs of age and an ideal transition to three race distances at 19yrs of age (male swimmers: 17 and 22yrs, respectively).

The literature reveals contradictory findings on the effect of skill variety, sampling of activities outside the domain sport, and transfer from junior to elite age success [3–6, 18–22]. In particular, the success transfer is heavily discussed in the swimming specific scientific literature. While some studies underlined the difficulties of transitioning success from junior to elite age [23], others studies showed that ranking and participation at Junior World Championships are positively related to success at senior World Championships [20]. Those contradictory findings may be due to the dose-time-effect of technical and physiological variety across a swimmer’s career. Rather than maximizing either variety or specialization, the present study shows that swimmers start their career with a larger variety but specialize at certain timepoints in the career to achieve later elite age success. Coaches should pay particular attention to the optimal transition points throughout the specialization process and to the most advantageous combinations of swimming strokes at peak performance age. If the trend in recent years of continuously improving world best times and increasing performance density in competitive swimming persists [24, 25], we may witness an even higher specialization throughout the coming years.

To increase variety yet prepare for the specific demand of swimming, the strategic implementation of other aquatic sports, i.e. water polo, underwater rugby, and competitive lifesaving, may help develop a broad range of motor skills, aerobic and anaerobic physiological capacity, as well as breath holding abilities [26, 27]. Additionally, with the increasing importance of the acyclic phases, i.e. starts and turns, across all race distances [28, 29], on-land weight-bearing sports during junior age may help develop leg strength, core stability, and postural control, which are important prerequisites for the strength and conditioning regimes including heavy weight lifting in later age categories [30, 31]. Previous studies showed that there actually is sufficient time in the development process of young swimmers to implement those alternative sports while allowing for specific training and competition routines [10]. Particular attention should be paid to sex differences regarding the implementation of alternative aquatic and land-based sports: due to earlier biological maturation [32], female compared to male swimmers showed earlier development of races times in a previous [15] and earlier specialization in the present study.

## Conclusion

In order to increase probability of becoming international-class, swimmers should optimize rather than maximize technical and physiological variety. As such, still competing in five swimming strokes towards elite age or specializing in only one swimming stroke right from the beginning of the career showed the lowest probabilities. Physiological variety increased during junior age and declined again towards elite age. Therefore, the dose-time-effect revealed by the present study specifies the optimal numbers of swimming strokes and race distances required at certain timepoints of the swimming career. While both sexes show similar specialization pattern throughout the development process, female swimmers specialize earlier than their male counterparts.

## Limitations

Although the study provides a representative sample of swimmers from 111 nations and swim races are the net outcome of a swimmer’s annual aspiration, races only present a fraction of the time that a swimmer spends in the water. Future studies will need to quantify technical and physiological variety in the training sessions of international-class swimmers, in order to quantify potential involvement in alternative aquatic and on-land sports as well. Additionally, the present study only quantified variety based on involvement in different swimming events. Assessment of the quality of variety, i.e. difference in race times between the swimmer’s main and other events, is a matter of future studies. It is worth noting that the dependent variable of the present study is ranking in 200 m swimming performance at peak performance age. The race time of about 2 min requires a balanced aerobic (66%) and anaerobic (34%) energy contribution [33] and may be adaptable to the needs of the neighboring race distances, i.e. 100 and 400 m. Sprint or long-distance swimmers may require greater and earlier specialization to develop the specific physiological and neuro-muscular abilities. Finally, the present data collection ended with the previous Olympic cycle, i.e. 2021. Recent developments such as the implementation of U23 European Championships may provide a transition period from junior to elite age competitions and may alter timepoints and extent of specialization and variety.

### Supplementary Information

**Additional file 1: Figure S1.** Probability (*p*) to become an international-class (A, C) female and (B, D) male *breaststroke *swimmer (>750 swimming points at peak performance age) when competing over different numbers of swimming strokes and race distances, respectively, across the age groups. The text boxes on the right end of the graphs show the most frequent combinations of swimming strokes and race distances at peak performance age. **Figure S2.** Probability (*p*) to become an international-class (A, C) female and (B, D) male *individual medley *swimmer (>750 swimming points at peak performance age) when competing over different numbers of swimming strokes and race distances, respectively, across the age groups. The text boxes on the right end of the graphs show the most frequent combinations of swimming strokes and race distances at peak performance age. **Figure S3.** Probability (*p*) to become an international-class (A, C) female and (B, D) male *backstroke *swimmer (>750 swimming points at peak performance age) when competing over different numbers of swimming strokes and race distances, respectively, across the age groups. The text boxes on the right end of the graphs show the most frequent combinations of swimming strokes and race distances at peak performance age. **Figure S4.** Probability (*p*) to become an international-class (A, C) female and (B, D) male *butterfly *swimmer (>750 swimming points at peak performance age) when competing over different numbers of swimming strokes and race distances, respectively, across the age groups. The text boxes on the right end of the graphs show the most frequent combinations of swimming strokes and race distances at peak performance age.

## Data Availability

Data are available on request by the corresponding author.

## Abbreviations

BU: Butterfly
BA: Backstroke
BR: Breaststroke
FR: Freestyle
IM: Individual medley
