# Supplementary material for: Variation vs. specialization: the dose-time-effect of technical and physiological variety in the development of elite swimmers
Source: BMC Res Notes. 2024 Feb 14;17:48. doi: 10.1186/s13104-024-06706-x (PMC10865614; doi:10.1186/s13104-024-06706-x)
Supplement: Supplementary file 1 — Additional file 1: Figure S1. Probability (p) to become an international-class (A, C) female and (B, D) male breaststroke swimmer (>750 swimming points at peak performance age) when competing over different numbers of swimming strokes and race distances, respectively, across the age groups. The text boxes on the right end of the graphs show the most frequent combinations of swimming strokes and race distances at peak performance age. Figure S2. Probability (p) to become an international-class (A, C) female and (B, D) male individual medley swimmer (>750 swimming points at peak performance age) when competing over different numbers of swimming strokes and race distances, respectively, across the age groups. The text boxes on the right end of the graphs show the most frequent combinations of swimming strokes and race distances at peak performance age. Figure S3. Probability (p) to become an international-class (A, C) female and (B, D) male backstroke swimmer (>750 swimming points at peak performance age) when competing over different numbers of swimming strokes and race distances, respectively, across the age groups. The text boxes on the right end of the graphs show the most frequent combinations of swimming strokes and race distances at peak performance age. Figure S4. Probability (p) to become an international-class (A, C) female and (B, D) male butterfly swimmer (>750 swimming points at peak performance age) when competing over different numbers of swimming strokes and race distances, respectively, across the age groups. The text boxes on the right end of the graphs show the most frequent combinations of swimming strokes and race distances at peak performance age. [file 13104_2024_6706_MOESM1_ESM.pdf]

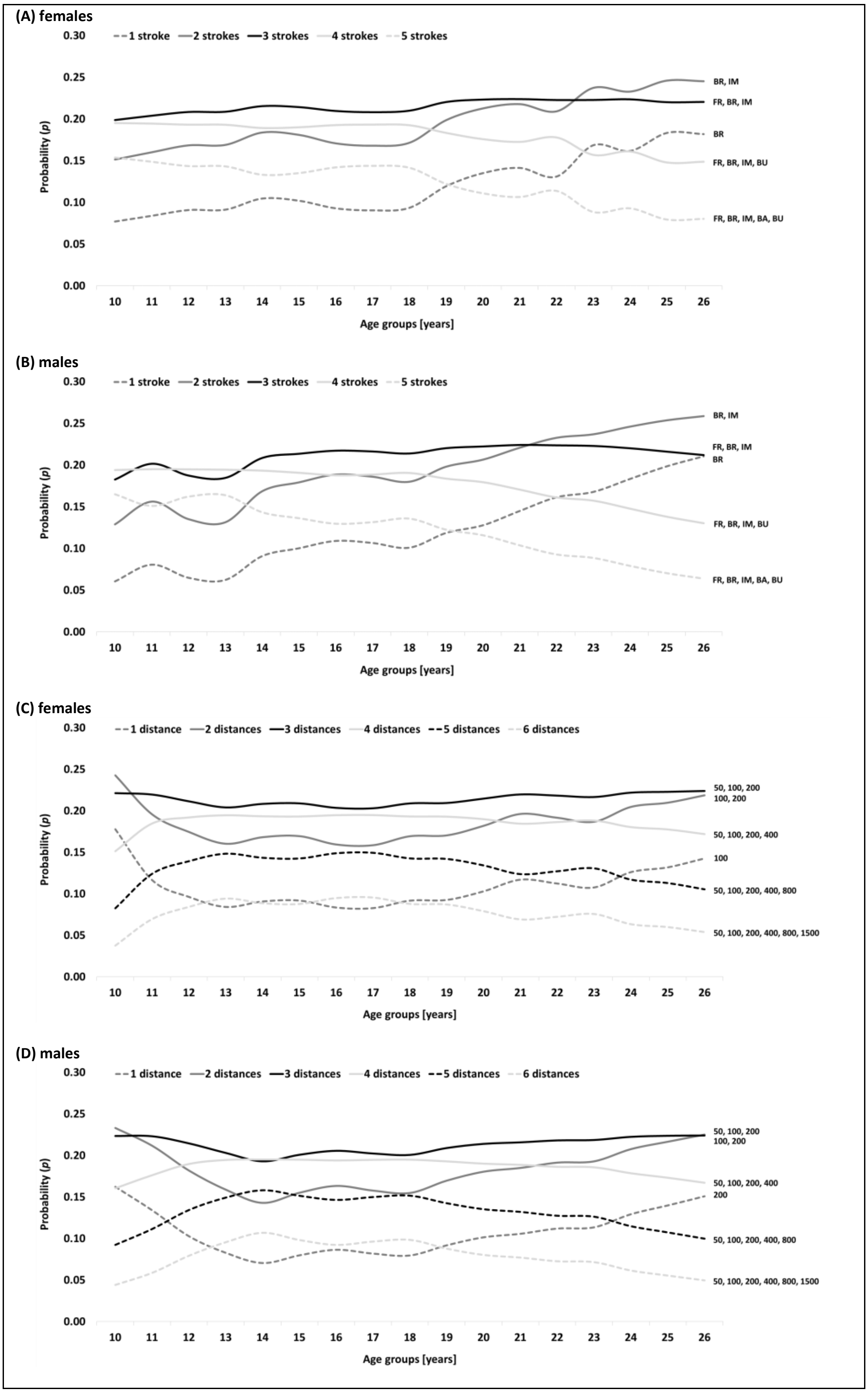

Figure A1 Probability (*p*) to become an international-class (A | C) female and (B | D) male ***breaststroke*** swimmer (>750 swimming points at peak performance age) when competing over different numbers of swimming strokes and race distances, respectively, across the age groups. The text boxes on the right end of the graphs show the most frequent combinations of swimming strokes and race distances at peak performance age.

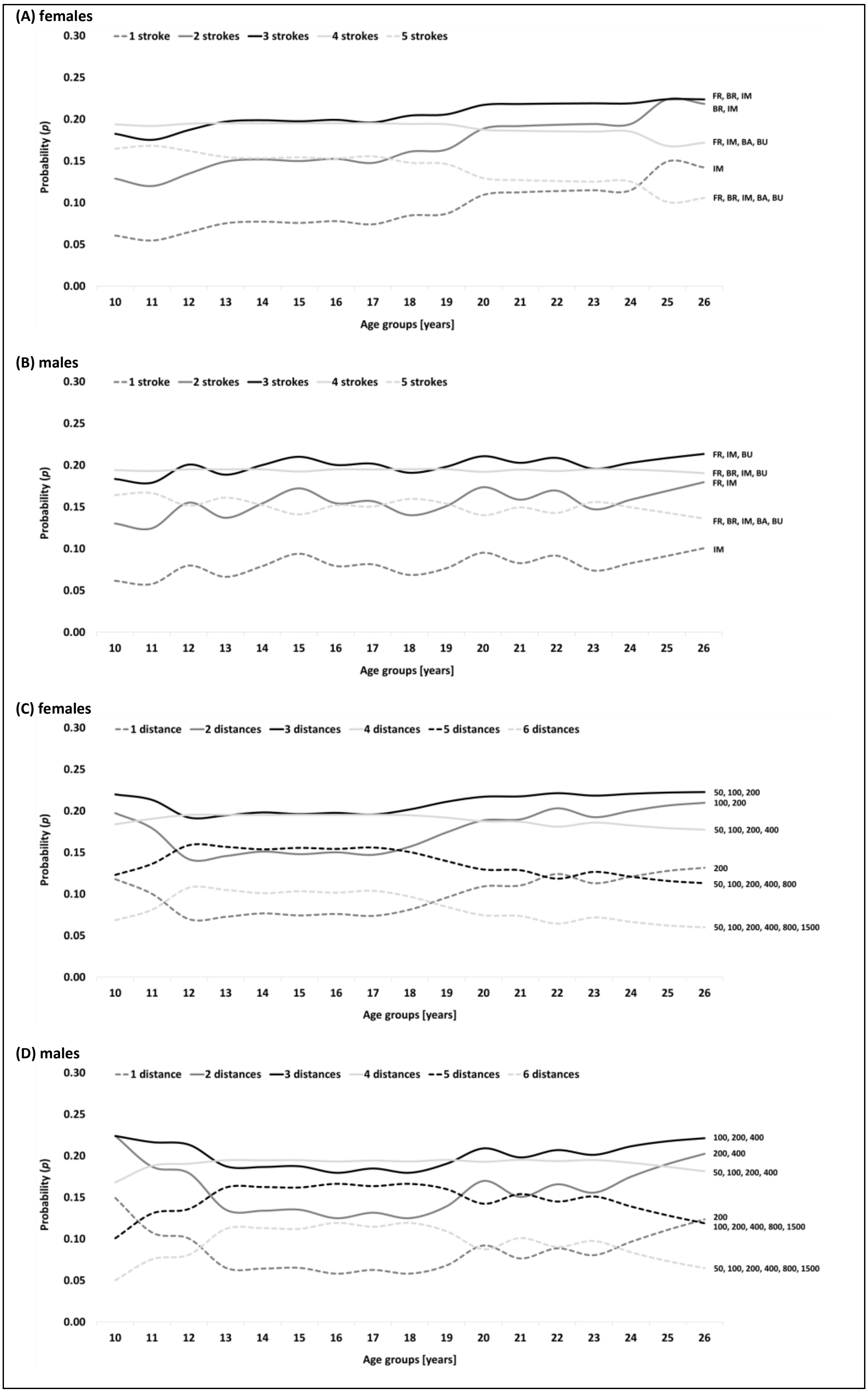

Figure A2 Probability (*p*) to become an international-class (A | C) female and (B | D) male ***individual medley*** swimmer (>750 swimming points at peak performance age) when competing over different numbers of swimming strokes and race distances, respectively, across the age groups. The text boxes on the right end of the graphs show the most frequent combinations of swimming strokes and race distances at peak performance age.

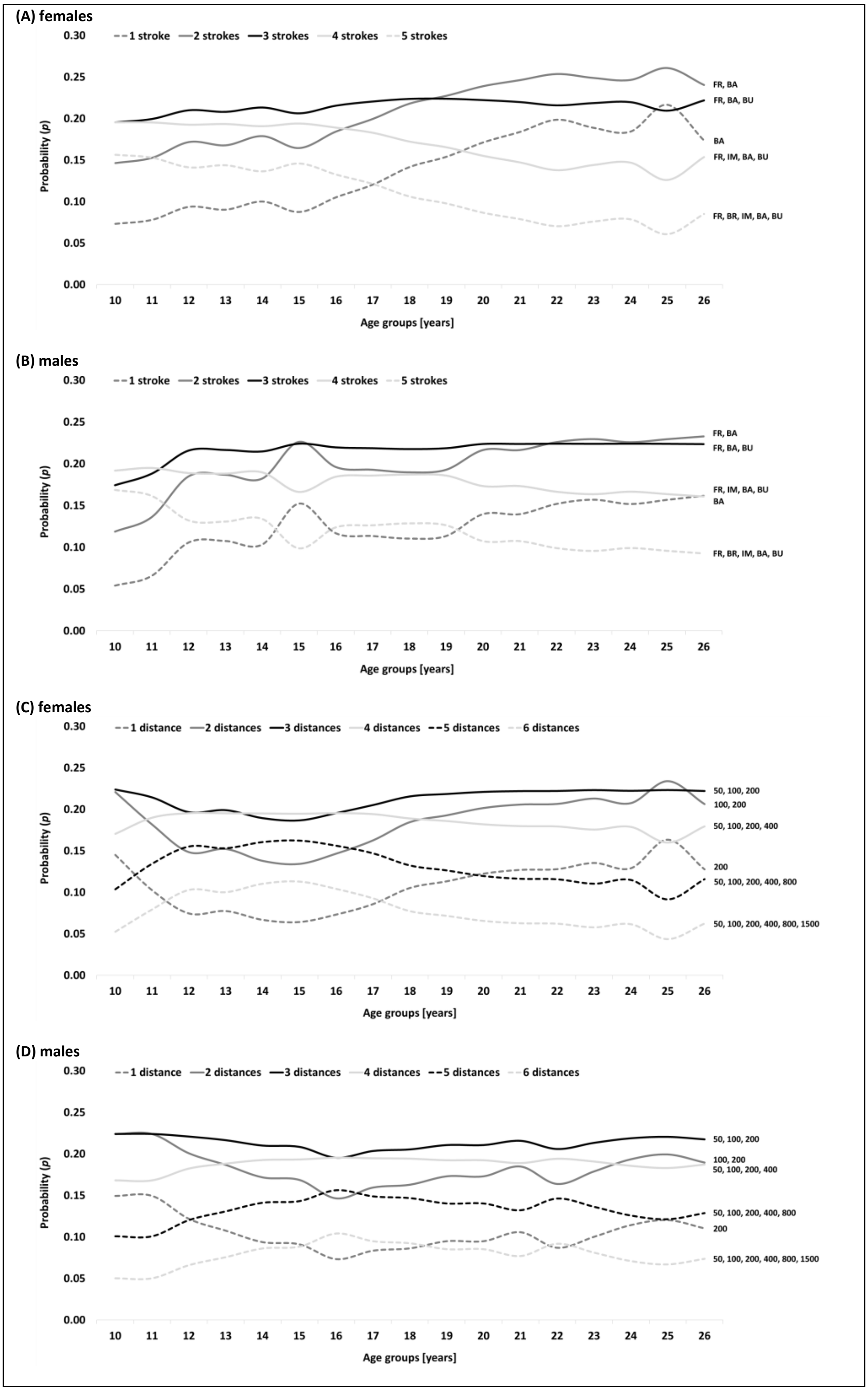

Figure A3 Probability (*p*) to become an international-class (A | C) female and (B | D) male ***backstroke*** swimmer (>750 swimming points at peak performance age) when competing over different numbers of swimming strokes and race distances, respectively, across the age groups. The text boxes on the right end of the graphs show the most frequent combinations of swimming strokes and race distances at peak performance age.

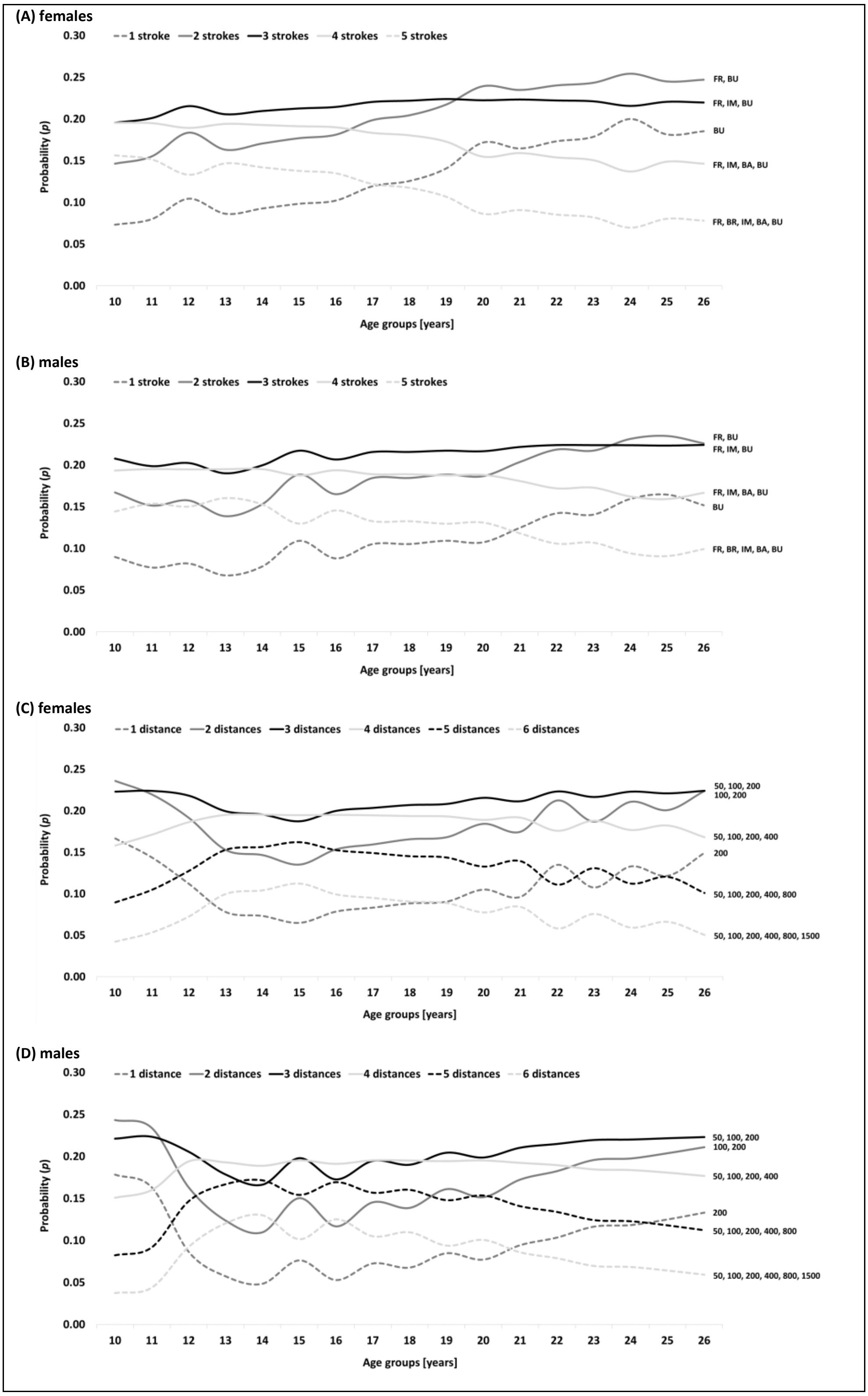

Figure A4 Probability (*p*) to become an international-class (A | C) female and (B | D) male ***butterfly*** swimmer (>750 swimming points at peak performance age) when competing over different numbers of swimming strokes and race distances, respectively, across the age groups. The text boxes on the right end of the graphs show the most frequent combinations of swimming strokes and race distances at peak performance age.
